# A methodological overview of new measure development for the Limb Injury Measurement Battery for Quality of Life (LIMB-QOL)

**DOI:** 10.1007/s11136-026-04298-6

**Published:** 2026-07-04

**Authors:** Aaron J. Boulton, Seung W. Choi, Callie E. Tyner, Jerry Slotkin, David S. Tulsky

**Affiliations:** 1 Center for Health Assessment Research and Translation, University of Delaware, 100 Discovery Blvd, Newark, DE 19713 USA; 2 Department of Educational Psychology, University of Texas at Austin, 1912 Speedway, Stop D5000, Austin, TX 78712 USA; 3 Departments of Physical Therapy and Psychological & Brain Sciences, University of Delaware, Newark, DE USA

**Keywords:** Health-related quality of life, Patient reported outcomes, Amputations, Traumatic, Upper extremity, Lower extremity, Outcome assessment, Health care

## Abstract

**Purpose:**

To describe the data analytic strategy used to develop new quality-of-life measures for the Limb Injury Measurement Battery for Quality of Life (LIMB-QOL).

**Methods:**

Several item pools were created and administered to a large sample of individuals with a history of major extremity injury or limb loss (n = 603). Item analyses adhered to modern psychometric standards (e.g., PROMIS®, COSMIN) and aimed to create several item response theory-based (IRT) item banks based on the graded response model. Items were removed iteratively based on pre-defined criteria and IRT model assumptions were met for the final item pools (monotonicity, unidimensionality, local item independence); differential item functioning, test–retest reliability, and convergent validity were then evaluated. Computer adaptive test and short-form versions of final item banks were created and examined using data simulation.

**Results:**

Item analyses led to the development of 8 new item banks and two fixed-length scales. These 10 new LIMB-QOL measures demonstrated initial evidence of reliability (α range = 0.94–0.98, test–retest ICC range: 0.68–0.91) and convergent validity for use in individuals with a history of major extremity injury or limb loss, and abbreviated formats of the full item banks exhibited comparable performance.

**Conclusion:**

The new LIMB-QOL measures demonstrated strong psychometric properties and can be used to collect patient-reported assessments of quality of life following major extremity injury and limb loss. The analytic strategy described herein exemplifies how the PROMIS methodology can be utilized to design IRT-based patient-reported outcome measures to fill measurement gaps for specific clinical populations.

**Supplementary Information:**

The online version contains supplementary material available at https://doi.org/10.1007/s11136-026-04298-6.

The Limb Injury Measurement Battery for Quality of Life (LIMB-QOL) is a comprehensive set of patient-reported outcome measures (PROMs) covering physical, emotional, and social domains that is tailored to individuals who have experienced one or more severe injuries to a major extremity and/or limb loss. LIMB-QOL was developed as part of a years-long effort to standardize and modernize PROMs for assessing health-related quality of life (HRQOL) in this population [1]. LIMB-QOL measures were built using item response theory (IRT) methods that underpin the most recent and widely used patient-reported outcomes systems, such as the Patient Reported Outcomes Measurement Information System [2] (PROMIS®) and Quality of Life in Neurological Disorders [3] (Neuro-QoL™). The measures were designed to broadly capture individuals’ HRQOL experiences following limb trauma across numerous domains identified as important by these patients and their clinicians [1, 4]. LIMB-QOL was designed to include existing item banks or scales that assess HRQOL constructs universally relevant across health populations, as well as newly developed, condition-specific measures of issues unique to severe limb injuries. To develop new item banks, item pools were constructed, analyzed, refined, and field tested in a large sample of individuals who experienced major extremity trauma or had sudden-onset illness that resulted in limb loss [1]. This paper describes the common analytic method used to develop and calibrate new IRT-based item banks, computer adaptive tests (CATs), and short forms.

Several features of the analytic methodology underscore the rigor with which the new LIMB-QOL measures were created and more broadly illustrate best practices for development of IRT-based HRQOL outcome measures. At each step of the process, multiple analytic techniques were utilized to thoroughly vet items individually and collectively. Both parametric and non-parametric techniques were used, in combination with several data visualizations, to distill core item sets for each measure. Emphasis was placed on removal of multidimensional items as well as those potentially biased against individuals with different injury presentations (e.g., limb loss vs. surgical preservation/reconstruction) or background characteristics (e.g., age). Once measures were finalized, we leveraged follow-up data and simulations to ensure item banks exhibited sufficient evidence of reliability/validity and established score equivalency between abbreviated formats (e.g., CAT) and item banks. We believe this structured and repetitious application of several interrelated psychometric and visualization methods enhanced our ability to identify poorly fitting items and develop new measures to fill critical measurement gaps for individuals with major extremity injury(s). This manuscript will thus serve as a methodological reference for several measure-specific articles to follow that delve more deeply into the development of each new LIMB-QOL instrument.

## Background

Development of LIMB-QOL followed a mixed-methods approach. Qualitative data were used to identify domains of functioning from which existing scales could be selected and new item banks could be developed to fill measurement gaps [1, 4]. Briefly, focus groups were conducted with individuals who had major limb trauma and subsequent limb amputation(s) or corresponding surgeries to preserve the limb(s). Several domains of functioning were identified, many of which referred to HRQOL issues for which item banks had previously been developed (e.g., fatigue). Additional domains were also identified for which there had not been systematic development of item banks, and are the focus of this manuscript. This work follows similar efforts to develop condition-specific measures in traumatic neurologic injury populations, such as the Spinal Cord Injury-Quality of Life [5] (SCI-QOL) and Traumatic Brain Injury-Quality of Life [6] (TBI-QOL) measurement systems.

In a manuscript describing an overview on the development of LIMB-QOL, Tulsky et al. [1] describe how item pools were created for these newly identified domains and procedures used for field-testing. Specifically, new item pools were administered along with existing measures to 603 individuals with confirmed major extremity injury or illness that resulted in either limb loss or the need for surgical limb preservation. Data from baseline assessments were used to develop the measures and anchor the scale to the target population. Follow-up analyses at 1- and 2- years were used for additional evaluations of reliability and validity. Item analyses described below adhered to field standards concerning modern, IRT-based PROM development (e.g., PROMIS scientific standards [7], COSMIN [8]). For the new item pools, we used the graded response model (GRM) [9] for analyses because it tends to fit ordered polytomous responses well, is widely researched and supported by current software, underpins many PROMs, and permits direct linking of scores on different measures of the same latent trait [10]. Moreover, new LIMB-QOL measures were developed as a set of separate, unidimensional measures that can be administered in a modular fashion to fit users’ needs; although multidimensional IRT models confer some efficiency advantages when administered as CATs [11], the simplicity, flexibility, and popularity of the unidimensional approach held greater appeal when creating LIMB-QOL.

## Data analysis methodology

### Overview

The LIMB-QOL item pools successfully finalized as item banks or scales are listed in Table 1. Additional information regarding the item pools is provided in the main LIMB-QOL development paper [1]. Analyses of the new item pools proceeded in five steps. All analyses were conducted in R (version 4.2.1); specific R packages used are provided in the Supplementary Material (Table S1). Select high-level results are presented throughout to illustrate key findings across the new measures; detailed results for each measure will be presented elsewhere.

**Table 1:** Item calibration results summary

Category	Domain	N	# Items	H (item *h*s)	α	ω_H_	ECV	χ^2^ (df)	RMSEA	CFI	SRMR
Physical Functioning	Satisfaction with Physical Fitness and Athleticism	602	15	0.55 (0.48–0.62)	0.95	0.87	0.80	558.293 (90)	0.094	0.991	0.056
	Satisfaction with Orthosis/Prosthesis	471	15	0.54 (0.41–0.62)	0.96	0.85	0.79	286.208 (90)	0.068	0.995	0.050
Emotional Health	Body Image	602	29	0.60 (0.42–0.69)	0.98	0.87	0.80	648.491 (348)	0.038	0.999	0.031
	Future Outlook	602	21	0.59 (0.47–0.69)	0.97	0.91	0.85	821.343 (189)	0.075	0.996	0.050
	Grief and Loss	602	25	0.66 (0.55–0.71)	0.98	0.89	0.85	757.290 (275)	0.054	0.998	0.039
	Resilience	603	40	0.50 (0.31–0.59)	0.98	0.90	0.84	2441.093 (740)	0.062	0.994	0.051
	Self-Esteem	602	34	0.61 (0.48–0.69)	0.98	0.86	0.79	2816.516 (527)	0.085	0.995	0.057
	Weight Satisfaction	602	10	0.65 (0.53–0.72)	0.95	0.85	0.77	229.863 (35)	0.096	0.996	0.057
	Health-Related Self-Efficacy	592	16	0.40 (0.29–0.46)	0.94	0.79	0.71	535.579 (104)	0.085	0.986	0.064
Social Participation	Vocational Impact	602	7	0.66 (0.60–0.72)	0.94	0.89	0.82	70.735 (14)	0.083	0.998	0.035

H, *h* = Loevinger’s test- and item-level H coefficient, respectively. α = internal consistency. ωH = omega hierarchical coefficient. ECV = explained common variance. **χ**2 (df) = Chi-square and degrees of freedom. RMSEA = Root Mean Square Error of Approximation. CFI = comparative fit index. SRMR = standardized root mean square residual

### Step 1: Item pool binning analysis

We first examined correlations between item pools to obtain a preliminary idea of the structure beneath the new measures as well as evaluate whether any pools might need to be collapsed. Sum scores were calculated for each item pool to compute correlations. Consistent with our hypotheses, the largest correlations were observed between Resilience and Future Outlook (*r* = 0.86) as well as between Self-Esteem and Body Image (*r* = 0.86). Self-Esteem was also strongly correlated with Resilience (*r* = 0.82) and Future Outlook (*r* = 0.80). Grief and Loss scores were strongly and negatively correlated with Self-Esteem (*r* = − 0.74) and Body Image (*r* = − 0.70). All other correlations between item pools ranged between − 0.70 and 0.70. Emotional domains of HRQOL are typically strong [12]; because the constructs were expected to narrow in scope during analyses, we did not collapse any pools prior to analysis.

### Step 2: Preliminary item analyses

**Fig. 1:**
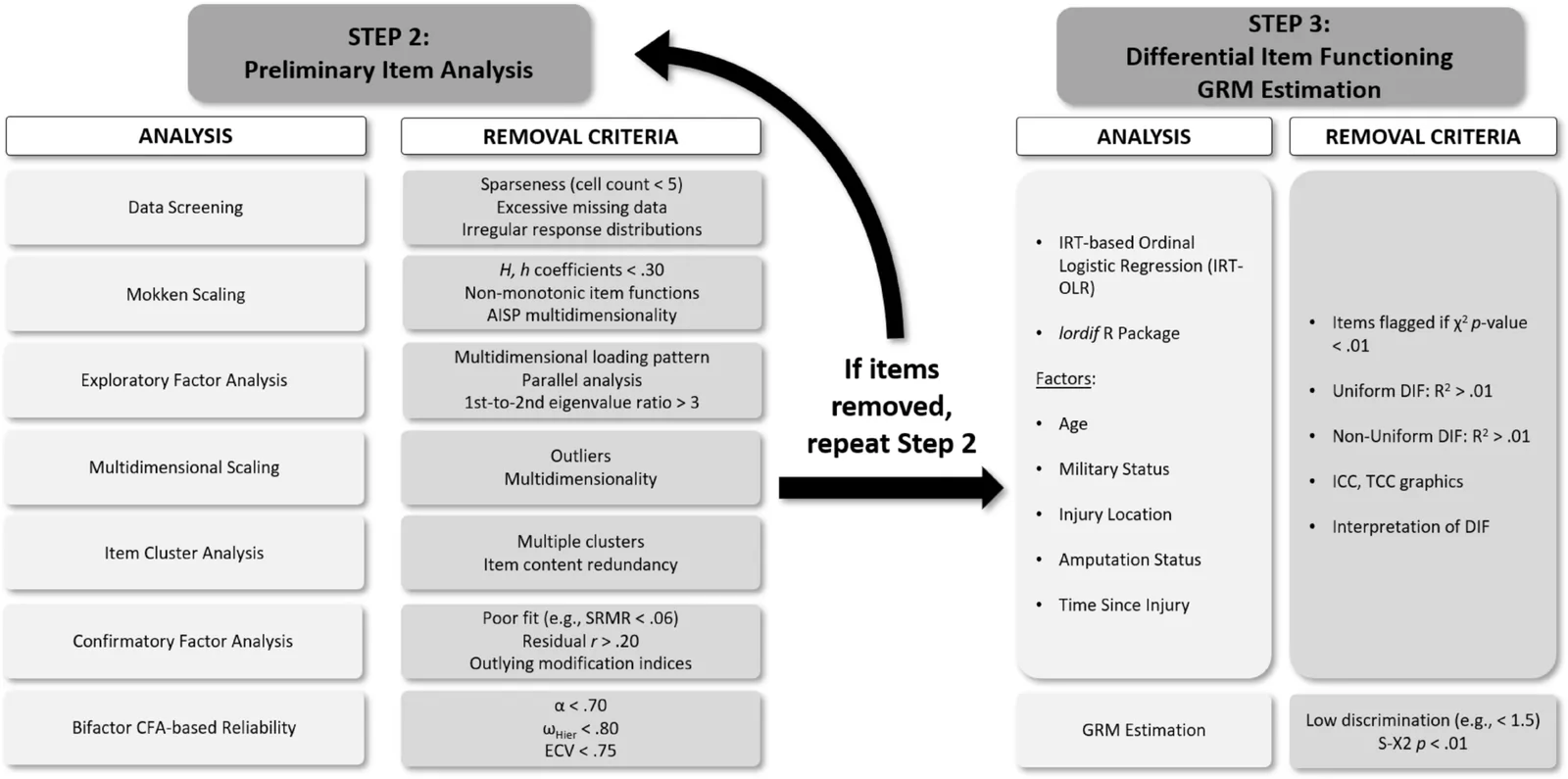
Item analysis methodology (Steps 2 and 3)

Analyses in Steps 2 and 3 were conducted iteratively for each item pool and constitute the item banking process; as a result, all analyses described below were conducted separately for each item pool during each analytic iteration. Figure 1 provides a summary of this process. Items that did not meet the pre-defined statistical criteria were flagged and typically removed, although in limited circumstances, items that did not satisfy all criteria were retained if item content was deemed essential and statistical violations were judged to be minor. Items exhibiting poor psychometric properties were often flagged across multiple approaches; this redundancy permitted a thorough and rigorous process for developing each bank. The research team held regular consensus meetings to review results and make determinations for each item. Items were removed incrementally (typically, 1–5 items were removed after each analytic iteration) until all remaining items satisfied criteria.

### Data screening

Response data were first screened for missing data, sparseness, and distributional patterns. Missing data rates were low (< 5% for all items tested); as a result, impact from missing data was considered negligible [13]. Moreover, the GRM was estimated using full information methods. Sparseness was also minimal; as such, we removed items with low cell counts as opposed to collapsing response categories, although a small number of items with cells containing < 5 responses that had otherwise acceptable psychometric properties were retained. Finally, item frequency distributions were computed during each analysis step to inform decision-making. Items with highly irregular patterns, such as multiple modal response categories, were flagged for potential removal.

### Evaluating IRT model assumptions

The prevailing assumptions of the GRM are monotonicity, unidimensionality, and local item independence [14]. The conceptual basis underlying each assumption and statistical procedures/criteria used for evaluation are described next.

### Monotonicity

**Fig. 2:**
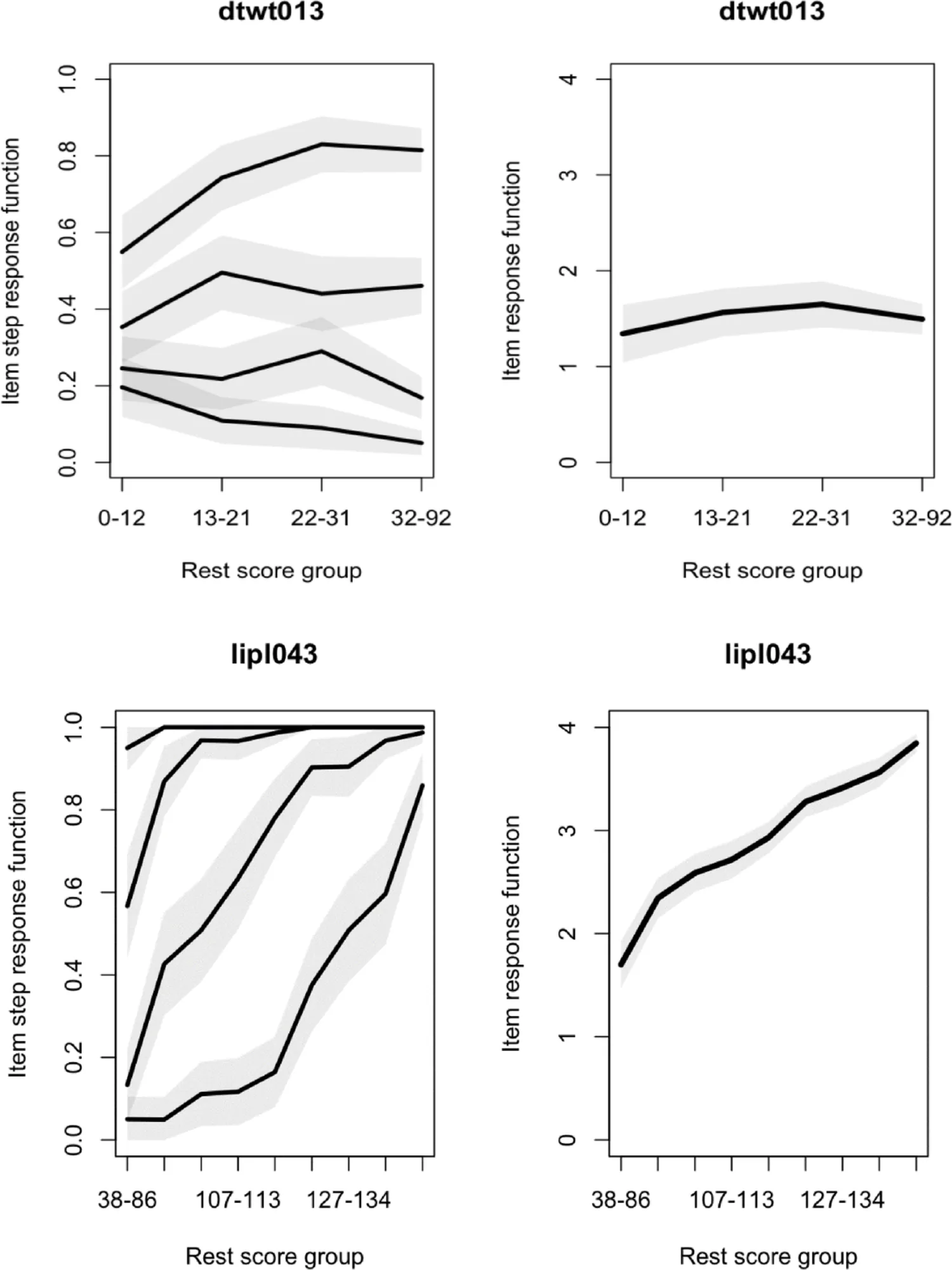
Rest score plots. In this figure, rest score plots are shown for two items (rows). The item dtwt013 (“I have exercised to manage my weight.”) clearly violates monotonicity, whereas the item lipl043 (“I believed I could accomplish anything I set my mind to.”) satisfies the assumption. The plots in the left column show the item response step functions, and the plots in the right-hand column show the item score function (referred to as the ‘item response function’ in the R package used for analyses). Confidence bands represent 95% confidence intervals

Under the GRM, it is assumed that the probability of endorsing a response category or any located above it strictly increases as scores on the latent trait increase. This *monotonicity* assumption was tested in the item pools using non-parametric Mokken Scaling [15, 16]. First, we visually evaluated monotonicity via rest score plots (Fig. 2). The first row in Fig. 2 reflects an item for which monotonicity was supported; the second shows a monotonicity violation. Rest scores, the sum of a set of items less one focal item, are plotted along the x-axis. In the left-side column, item response step functions (IRSFs) are plotted, which represent the probability of endorsing option *x* or higher; there are four IRSFs for a 5-response choice option item. In the right-hand column, an item score function is plotted, which represents the expected item score for each rest score group. In both plots, functions are expected to strictly increase as rest scores increase, although minor violations can occur and did not necessarily lead to item removal.

Two other Mokken scaling procedures were used: Loevinger’s scalability coefficient *H* [17] and the Automated Item Selection Procedure [15] (AISP). Neither is a direct test of monotonicity, but both are considered part of the Mokken scaling toolkit and items that violate monotonicity also tend to show poor performance under both procedures. The H coefficient (range: 0–1), is defined at the item and test levels and reflects the extent to which an item or test accurately orders or discriminates among respondents [16]. Conventionally, H coefficients lower than 0.3 indicate “unscalable” items/tests; values of H between 0.3 and 0.4 reflect “weak” scalability, between 0.4 and 0.5 “medium” scalability, and greater than 0.5 “strong” scalability [18]. Coefficients for the final item banks and scales are shown in Table 1. The AISP procedure uses H coefficients to select one or more subsets of scalable and/or non-scalable items and is more closely related to scale unidimensionality than monotonicity. When most or all items were selected for the first subset, unidimensionality was supported.

### Unidimensionality

The development of multiple *unidimensional* outcome measures was previously stated as a core objective of the LIMB-QOL study. Unidimensionality is a key assumption of the GRM. Multiple descriptive, graphical, and inferential procedures were used, as no gold standard exists for assessing unidimensionality, and research suggests advantages to using a combination of approaches [19].

*Exploratory Factor Analyses (EFA)* EFA was used to identify items that might contradict unidimensionality. A single EFA was fit, extracting three factors using principal axis factoring followed by an oblimin rotation. Inspection of loadings and factor correlations signaled whether the factor(s) reflecting the general factor–typically the first factor, although in some cases the first two or three factors, if highly correlated–contained sufficiently high loadings and/or whether any items exhibited salient secondary loadings. Parallel analyses [20] were also conducted, as well as computation of first-to-second eigenvalue ratios (ideally > 3 [21]).

**Fig. 3:**
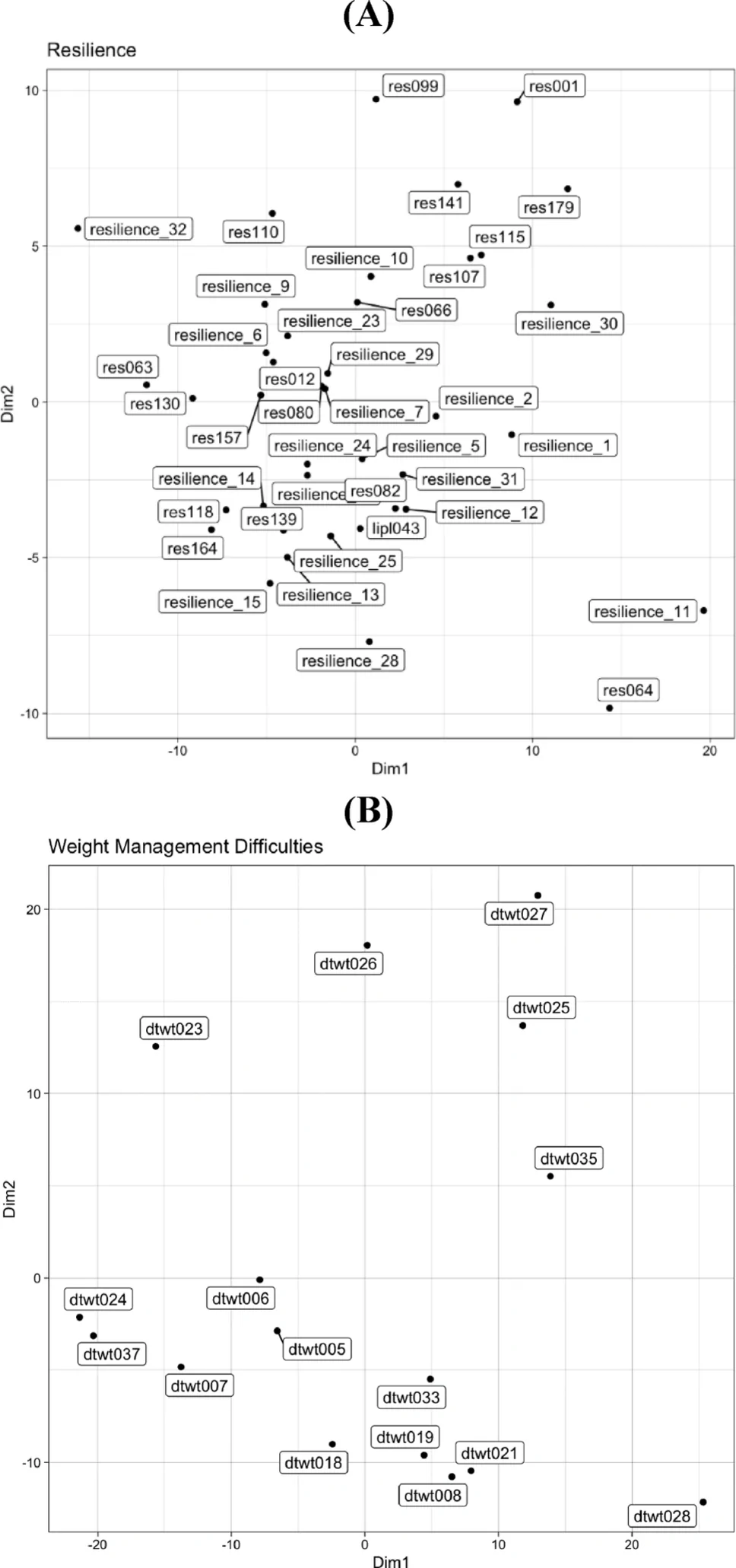
MDS Plots. In the plots, items are projected into a two-dimensional space. The Resilience item pool (**A**) exhibited strong unidimensionality as indicated by the clustering of items. For the Weight Management Difficulties item pool (**B**), multidimensionality was present, leading to a split in the item pool

*Multidimensional Scaling* Multidimensional scaling (MDS) plots [22] were also used to visualize item pool dimensionality. Items were projected into two-dimensional space representing Euclidean distances between items. In Fig. 3, two exemplar MDS plots are shown. Figure 3A shows the final 40-item Resilience item bank [23], which exhibited strong unidimensionality as evidenced by clustering of items. Outlying items, which usually exhibited weaker relations with other items (e.g., resilience_11 in Fig. 3), were often candidates for removal. Figure 3B–obtained from the second analytic iteration of the Weight Management Difficulties item pool–exemplifies the multidimensional case, in which two distinct clusters of items (along the y-axis) were observed. This item pool was later split into separate pools based on item content [24].

*Item Cluster Analysis* Item cluster analysis [25], a form of hierarchical cluster analysis performed on items instead of cases, was also used to visualize dimensionality. The primary output is a dendrogram that displays the order in which items merge into a smaller number of clusters. Unidimensionality is supported when all items merge into one cluster, though in practice this was not necessarily sufficient for proceeding to the next analytic step; items that joined the main cluster late in the process typically exhibited weaker relations with other items. Conversely, items first to pair with other items often reflected content redundancies.

*Confirmatory Factor Analysis* Confirmatory factor analysis (CFA) models estimated via diagonally-weight least squares (DWLS) estimation were used to determine whether a one-factor model provided acceptable fit. Conventional SEM-based alternative fit indices and cutoff values were examined–Root Mean Square Error of Approximation [26] (RMSEA) < 0.08, Comparative Fit Index [27] (CFI) and Tucker-Lewis Index [28] (TLI) > 0.90, and Standardized Root Mean Square Residual [29] (SRMR) < 0.06 were considered acceptable [7, 30–32]–although we did not exclusively rely on these thresholds for decision-making, given their well-known shortcomings [33, 34]. For instance, CFI and TLI values can often be inflated in categorical factor analysis models [35], which may lead to under-identification of model misfit, whereas the RMSEA can be inflated when examining shorter forms [36], which may lead to over-identification of negligible model misfit. Other CFA results, including factor loadings, residual correlations, and modification indices, were also used to guide decision-making.

*Bifactor CFA* Bifactor CFA models were estimated to further explore dimensionality [37]. Because group factor structures were in most cases unknown a priori, bifactor models were estimated using an EFA-based procedure implemented in the omega function of the R *psych* package [25]. Models with one general and three group factors were estimated, which is the minimum number of factors necessary for bifactor model identification [21], and considered sufficient to capture salient secondary dimensions. In addition to internal consistency, which was estimated as part of these analyses–α values > 0.70 were considered acceptable–two indices based on the bifactor model were examined: Omega hierarchical [38] (ω_H_) and explained common variance [39] (ECV). The former quantifies the amount of variance in the sum of items attributable to the general factor, whereas the latter quantifies common variance attributable to the general factor. The ECV is theoretically more appropriate for assessing whether a set of items is “unidimensional enough” [40], although both have shown acceptable performance in simulations. ECV values > 0.75 and ω_H_ values > 0.80 were considered acceptable [41]. As shown in Table 1, all new LIMB-QOL banks and scales met these criteria except for the Health-Related Self-Efficacy item bank [42], although other evidence supported this measure.

### Local item independence

Local item independence in a unidimensional IRT model refers to the ideal case in which all covariation among items is accounted for by the latent trait. Excess local dependence (LD) can blur the meaning of scores in a similar manner to that of multidimensionality [43]. Two strategies were used to identify violations of LD. First, pairwise residual correlations from the single-factor CFA models were inspected; items exhibiting correlations greater than 0.20 [44] were candidates for removal. Modification indices (MIs) from the CFAs were also inspected; although any MI value greater than 3.84 represented a statistically significant improvement in fit (chi-square with 1 degree of freedom at α = 0.05), we instead focused on outlying values to flag instances of LD. Typically, item pairs with residual correlations greater than 0.20 also exhibited outlying MI values; LD could also be identified graphically in the MDS (close proximity) and item cluster analysis (early clustering, large correlations) plots. In general, item pools contained at least one item–though in some cases several items–that exhibited LD and were removed; details regarding specific items are to be reported elsewhere.

### Step 3: Differential Item Functioning (DIF) and item calibration

Once all psychometric criteria from Step 2 were met, the remaining items in a pool were tested for DIF. DIF tests were conducted for each of 5 grouping variables: Age (< 40 vs. 40+), military status (civilian vs. active-duty/reservist/veteran), injury location (lower limb only vs. upper limb/both upper and lower limbs), amputation status (any major extremity amputated due to injury vs. no amputations), and time since injury (< 3 years vs. 3 + years). The grouping factors were chosen based on theoretical and practical importance as well as the need for sufficient sample sizes within each subgroup (we aimed for at least 200 participants per subgroup). The IRT-based ordinal logistic regression (OLR) method was used [45], which involves comparing the fit of a series of nested models mirroring different levels of DIF (no DIF, uniform DIF, non-uniform DIF). Items were flagged if the difference in model fit values was significant at 0.01, or the difference in model pseudo R^2^ values was greater than 0.01; these values were intended to be conservative, as they are more sensitive than published guidelines [46]. However, we wished to ensure that even if DIF were negligible for many items, the cumulative effect was not impactful at the scale level [47]. If items were removed due to evidence of DIF, all preliminary item analyses were re-run to ensure the revised item pool still supported GRM assumptions. In general, only a few items (at most 3) were removed from each item pool due to DIF except in one instance: the Satisfaction with Orthosis and Prosthesis item pool contained several items that exhibited DIF due to amputation status (i.e., surgical preservation vs. amputation) due to the different types of devices worn by most individuals in each subcategory [48]. Nevertheless, the final 15-item version of this measure did not exhibit any remaining evidence of DIF. Following the DIF analyses, final GRM parameters were estimated, and results evaluated by inspecting category response functions, test- and item-level information functions, and item fit statistics (S-X2 [49, 50]). For any items flagged for S-X2 (*p* < 0.01), we additionally verified that their empirical item response curves closely aligned with the theoretical curves implied by the estimated item parameters.

### Step 4: CAT and short form development

The advantages of item banking of health outcome measures and use of calibration data to guide the selection of items are well-documented [51]. Chief among these is a gain in “measurement efficiency” such that respondent burden can be meaningfully reduced with minimal loss in–or even improvement in [52]—an instrument’s psychometric properties or clinical utility. Moreover, selection of item subsets from the overall bank–via CAT or by end users to create customized short forms–allows tailoring of the instrument to the measurement context.

LIMB-QOL CAT performance was initially tested in data simulations for each item bank to examine item usage and ensure score estimation was accurate under widely used stopping rules (e.g., < 0.30 standard errors, 4–8 item minimum/maximum). Three CAT specifications, as well as a short form, were tested using the calibration sample data: (1) A fixed-length 4-item minimum CAT, representing brief bank administration; (2) A *k*-fixed length CAT, where *k* was the number of items comprising the initial short form for the item bank, thus representing a comparison between the short form and a CAT of similar length; and (3) A 4-item minimum, 8-item maximum length CAT, representing a commonly used CAT specification. Correlations between the full item bank and abbreviated format scores were computed (*r*s were greater than 0.90 in all cases), and descriptive summaries of the score distributions compared (means and SDs were nearly identical across all formats). Score reliabilities were also compared graphically across latent trait continua. Detailed results of these comparisons can be found in companion manuscripts for individual measures. In Table 2, correlations between the 10 new LIMB-QOL measures computed at baseline are reported; correlations between the new measures and existing measures included in LIMB-QOL are provided in Supplemental Material (Table Sl).

**Table 2:** Correlations between LIMB-QOL item banks/scales

Domain	Satisfaction with physical fitness and athleticism	Satisfaction with orthosis/prosthesis	Body image	Future outlook	Grief and loss	Resilience	Self-esteem	Weight satisfaction	Health-related self-efficacy	Vocational impact
Satisfaction with physical fitness and athleticism		471	602	602	602	602	602	602	592	602
Satisfaction with orthosis /prosthesis	0.43 [0.35, 0.50]		471	471	471	471	471	471	471	471
Body image	0.69 [0.65, 0.73]	0.38 [0.30, 0.45]		602	602	602	602	602	592	602
Future outlook	0.55 [0.49, 0.60]	0.29 [0.20, 0.37]	0.64 [0.59, 0.69]		602	602	602	602	592	602
Grief and loss	− 0.70 [− 0.73, − 0.65]	− 0.36 [− 0.44, − 0.28]	− 0.74 [− 0.77, − 0.70]	− 0.61 [− 0.66, − 0.56]		602	602	602	592	602
Resilience	0.59 [0.53, 0.64]	0.30 [0.22, 0.38]	0.65 [0.60, 0.69]	0.86 [0.84, 0.88]	− 0.64 [− 0.69, − 0.59]		602	602	592	602
Self-esteem	0.68 [0.64, 0.72]	0.34 [0.26, 0.42]	0.85 [0.83, 0.87]	0.79 [0.76, 0.79]	− 0.78 [− 0.81, − 0.74]	0.82 [0.79, 0.85]		602	592	602
Weight satisfaction	0.55 [0.49, 0.60]	0.25 [0.16, 0.33]	0.61 [0.56, 0.66]	0.43 [0.36, 0.49]	− 0.38 [− 0.44, − 0.31]	0.43 [0.37, 0.50]	0.51 [0.44, 0.56]		592	602
Health-related self-efficacy	0.29 [0.21, 0.36]	0.29 [0.21, 0.37]	0.39 [0.32, 0.46]	0.52 [0.46, 0.58]	− 0.34 [− 0.41, − 0.27]	0.54 [0.48, 0.60]	0.49 [0.42, 0.55]	0.31 [0.24, 0.38]		602
Vocational impact	− 0.62 [− 0.67, − 0.57]	− 0.44 [− 0.51, − 0.36]	− 0.54 [− 0.59, − 0.48]	0.53 [− 0.58, − 0.47]	0.63 [0.58, 0.68]	− 0.48 [− 0.54, − 0.42]	− 0.57 [− 0.62, − 0.51]	− 0.33 [− 0.40, − 0.26]	− 0.26 [− 0.34, − 0.19]	

Correlations and 95% confidence intervals are shown below the diagonal. Pairwise sample sizes used for computation are shown above the diagonal

### Step 5: Test–retest reliability and convergent validity

Test–retest reliability and convergent validity were evaluated at the 1-year follow-up assessment. Participants completed items from the new item pools as well as convergent validity measures using the same interview format from baseline; at least one validity measure was administered for each new item pool. For test–retest analyses, a second interview took place 1–2 weeks later, with participants completing LIMB-QOL measures only. To reduce participant burden, most criterion measures were administered in a block randomized format. Measures were assigned to one of two blocks, and blocks were randomly assigned so that each participant completed only a single block. A small number of measures were not randomized but instead administered to all individuals meeting some condition (e.g., current/former member of military; assistive device user) to meet analysis sample size targets. To reduce burden even further, short forms were administered for the new measures [1]. Test–retest reliability was estimated via the intraclass correlation coefficient (ICC), with lower bound 95% confidence interval values greater than 0.75 or 0.90 deemed acceptable or excellent, respectively. For 9 of the 10 new measures, ICCs ranged between 0.77 and 0.91; although the ICC was 0.68 for Health-Related Self-Efficacy, other psychometric properties of this measure were strong, and given the scale is not intended for high-stakes decision-making (e.g., individual health care decisions), the measure was considered acceptable. Convergent validity was assessed via Pearson correlations between each new LIMB-QOL measure and associated criterion measure(s); values greater than 0.50 were often considered sufficient evidence of convergent validity, though target thresholds varied in some cases as a result of measure-specific hypotheses, as will be reported elsewhere.

## Discussion

The primary goal of creating LIMB-QOL was to provide the field with a set of standardized and validated assessment tools to track the most important HRQOL issues for those who have suffered the life-altering effects of limb loss or major extremity injury. Significant progress has been made in the last 15–20 years by PROMIS investigators and others to develop modern, IRT-based PROMs for the assessment of universal symptoms and functional domains, many of which are central to those with major extremity injuries. Although these tools can form the foundation for clinical and research applications, issues were identified by major extremity injury and limb-loss stakeholders [4] that warranted additional item banks to fill measurement gaps. This led the research team to develop new item banks and scales using a rigorous, multifaceted approach that had been used to construct measures for other rehabilitation populations [5, 6].

In this article, we have provided a detailed description of how the new item pools were analyzed and calibrated as 8 item banks and 2 fixed-length scales within the IRT framework. A systematic and rigorous procedure was used to verify GRM assumptions prior to estimation of GRM item parameters. Subsequent tests of DIF, test–retest reliability, and convergent validity in accordance with modern PROM scientific standards, as well as the development and simulation-based tests of CAT and short form versions of each item bank, further underscores the rigor of the analysis strategy described herein. The most vital feature of the item analytic approach, however, was the integration and extensive discussion of all quantitative information in combination with clinical and translational considerations at regular analysis meetings held by the research team. Overall, we believe that this methodological approach is detailed, rigorous, and comprehensive, and can serve as a valuable reference for future studies on the development of HRQOL measures.

### Study limitations and future directions

Development and release of a new PROM is only the first step in the much longer and ongoing process of evaluation, adoption, and refinement necessary for an instrument to impact in patient care and HRQOL research. As such, future research is needed to further evaluate and support use of the 10 new LIMB-QOL measures, including, for instance, additional tests of external validity (e.g., responsiveness), the factor structure of the overall battery, and trajectories of symptoms, especially in the immediate post-injury period. Creation of composite or clinical cut scores could further enhance the utility of these measures in a variety of settings.

Some limitations were encountered when developing the new item banks and scales. First, unbalanced sample sizes precluded some DIF comparisons; for instance, we could not compare item parameters across males and females due to the limited number of female participants enrolled in the study. Second, our sample reflects a primarily chronic sample of individuals with a wide array of limb-threatening injuries and illnesses, including both participants with and without limb loss, traumatic and non-traumatic injuries, and single as well as multiple affected limbs.

## Supplementary Information

Below is the link to the electronic supplementary material.

Supplementary Material 1

## Data Availability

Once the complete set of primary analyses/manuscripts from this study have been published, the de-identified quantitative data that support the findings of this study will be available from the corresponding author, DT, upon reasonable request. Qualitative data will not be made available due to the potential for confidentiality concerns.
