# Supplementary material for: A methodological overview of new measure development for the Limb Injury Measurement Battery for Quality of Life (LIMB-QOL)
Source: Qual Life Res. 2026 Jul 4;35(8):223. doi: 10.1007/s11136-026-04298-6 (PMC13332956; doi:10.1007/s11136-026-04298-6)
Supplement: Supplementary file 1 — Supplementary Material 1 [file 11136_2026_4298_MOESM1_ESM.docx]

**Supplementary Material**

In the tables below, we present (a) a list of R packages used for analyses, and (b) correlations between the newly created and existing measures that constitute the Limb Injury Measurement Battery for Quality of Life (LIMB-QOL). More detail regarding LIMB-QOL can be found in the LIMB-QOL introductory overview article cited in the main manuscript.

**Table S1. R Packages Used for Analyses**

| **Analysis** | **R package** |
| --- | --- |
| Mokken Scaling | mokken |
| Exploratory Factor Analysis | psych |
| Multidimensional Scaling | stats ^a^ |
| Item Cluster Analysis | psych |
| Confirmatory Factor Analysis (CFA) | lavaan |
| Bifactor CFA | psych |
| Differential Item Functioning (DIF) | lordif |
| GRM Estimation | mirt |

*Note*. ^a^ The *stats* package is included in the base R distributions. Additional information regarding R packages can be found at https://cran.r-project.org/.

**Table S2. Correlations between New and Existing Measures in LIMB-QOL**

|  | Body Image | Resilience | Future Outlook | Grief & Loss | Self-Esteem | Satisfaction with Orthosis/Prosthesis | Vocational Impact | Satisfaction with Physical Fitness & Athleticism | Health-Related Self-Efficacy | Weight Satisfaction |
| --- | --- | --- | --- | --- | --- | --- | --- | --- | --- | --- |
| Pain Interference  (PROMIS v1.0) | -.43 | -.39 | -.35 | .50 | -.43 | -.33 | .51 | -.58 | -.17 | -.31 |
| Pain Intensity  (PROMIS v1.0) | -.31 | -.27 | -.23 | .37 | -.29 | -.31 | .45 | -.43 | -.12 | -.25 |
| Fatigue  (PROMIS v1.0) | -.54 | -.53 | -.50 | .58 | -.59 | -.26 | .49 | -.59 | -.33 | -.43 |
| Independence  (SCI-QOL and TBI-QOL) | .62 | .52 | .51 | -.64 | .65 | .46 | -.56 | .67 | .26 | .37 |
| Lower Extremity Function/Mobility  (Neuro-QoL v1.0) | .39 | .32 | .31 | -.44 | .39 | .40 | -.50 | .55 | .18 | .33 |
| Fine Motor  (SCI-FI/C) | .21 | .04 | .08 | -.20 | .15 | .27 | -.27 | .17 | .08 | .14 |
| Self-Care  (SCI-FI/C) | .31 | .18 | .20 | -.32 | .27 | .37 | -.40 | .36 | .17 | .24 |
| Depression  (PROMIS v1.0) | -.61 | -.65 | -.62 | .67 | -.71 | -.32 | .52 | -.53 | -.38 | -.41 |
| Anxiety  (PROMIS v1.0) | -.60 | -.57 | -.54 | .63 | -.65 | -.29 | .45 | -.49 | -.35 | -.38 |
| Anger  (PROMIS Item Bank v1.1) ^b^ | -.50 | -.52 | -.49 | .58 | -.57 | -.23 | .43 | -.44 | -.35 | -.33 |
| Positive Affect and Well-Being  (Neuro-QoL v1.0) | .62 | .86 | .86 | -.61 | .79 | .28 | -.47 | .52 | .51 | .43 |
| Ability to Participate in SRA  (Neuro-QoL v1.0) | .62 | .59 | .57 | -.61 | .68 | .44 | -.59 | .67 | .42 | .41 |
| Satisfaction with SRA  (Neuro-QoL v1.0) | .66 | .65 | .61 | -.65 | .71 | .43 | -.58 | .75 | .38 | .45 |
| Stigma  (SCI-QOL) | -.73 | -.48 | -.48 | .69 | -.67 | -.32 | .52 | -.56 | -.26 | -.38 |
| Economic Quality of Life v2.0 ^a^ | .30 | .26 | .32 | -.34 | .28 | .27 | -.29 | .25 | .28 | .20 |

*Notes*: Neuro-QoL = Quality of Life in Neurological Disorders. PROMIS = Patient-Reported Outcomes Measurement Information System. SCI-FI/C = Spinal Cord Injury-Functional Index, Capacity. SCI-QOL = Spinal Cord Injury-Quality of Life. SRA = Social Roles and Activities. TBI-QOL = Traumatic Brain Injury-Quality of Life. Measure source provided in parentheses.
